# Clinical and histopathological features of chronic hepatitis B with normal alanine aminotransferase levels with or without concomitant non-alcoholic fatty liver disease

**DOI:** 10.3389/fmed.2026.1626154

**Published:** 2026-02-12

**Authors:** Wencong Li, Shiheng Liu, Weiguang Ren, Xiaoxiao Zhang, Lingdi Liu, Fang Han, Ying Zhang, Yuemin Nan, Suxian Zhao

**Affiliations:** Department of Traditional and Western Medical Hepatology, Hebei Medical University Third Hospital, the Key Laboratory of Hepatic Fibrosis Mechanisms of Chronic Liver Diseases in Hebei Province, HebeiInternational Science and Technology Cooperation Base - Hebei International Joint Research Center for Molecular Diagnosis of Liver Cancer, Shijiazhuang, China

**Keywords:** alanine aminotransferase, chronic hepatitis B, HBcAg, non-alcoholic fatty liver disease, pathology

## Abstract

**Objective:**

To analyze the clinical and hepatic pathological characteristics of patients with chronic hepatitis B (CHB) alone or with non-alcoholic fatty liver disease (NAFLD), who have normal alanine aminotransferase (ALT) levels.

**Methods:**

The patients with normal ALT levels and pathologically diagnosed CHB alone or in combination with NAFLD were enrolled, the demographic, laboratory, and pathological data were collected and analyzed.

**Results:**

Among 391 enrolled CHB patients with normal ALT levels, 107 individuals combined with NAFLD. The incidence of significant liver injury (G and/or S ≥ 2) in patients with CHB alone was lower significantly than that of patients with CHB and NAFLD (64.08% vs. 78.50%, *P* < 0.05), especially by the subgroup analyses in HBeAg positive, male, age > 30 years (*P* < 0.05). In all patients, who with negative HBcAg expression had lower HBV DNA levels than those with positive HBcAg expression (*P* < 0.001). Patients with G and/or S ≥ 2 had a higher proportion of HBcAg cytoplasm/cytoplasmic nucleus expression type compared to patients with liver tissue G and S < 2 (32.42% vs. 16.67% in CHB alone, 36.91% vs. 21.74% in CHB + NAFLD, all *P* < 0.001). Aspartate aminotransferase to ALT ratio index (AAR) demonstrated relatively superior efficacy in diagnosing significant liver fibrosis among patients with CHB and NAFLD, while fibrosis-4 (FIB-4) exhibited superior performance in patients with CHB alone.

**Conclusion:**

Patients with normal ALT can still demonstrate significant liver tissue damage. The combination of NAFLD will significantly increase the incidence of significant liver injury in patients with CHB, especially in those who are HBeAg positive, male, over 30 years old.

## 1 Introduction

Hepatitis B virus (HBV) infection represents a significant global health concern. In 2019, an estimated 316 million chronic hepatitis B (CHB) infections were reported worldwide (1). CHB can maintain the liver in a prolonged state of chronic inflammation, potentially leading to cirrhosis and hepatocellular carcinoma (HCC). Consequently, active and effective antiviral therapy capable of inhibiting viral replication and reducing hepatic inflammation is crucial for preventing or delaying liver cirrhosis and decreasing the incidence of HCC. Alanine aminotransferase (ALT) is an easily accessible surrogate marker for the presence or absence of disease activity within the liver (2). Currently, ALT serves as the most sensitive and direct indicator of liver tissue damage, making it a fundamental criterion for initiating antiviral therapy in CHB patients. However, recent studies have revealed that a substantial proportion of patients exhibit significant liver injury even with normal ALT levels (3, 4). Therefore, analyzing the clinical and hepatic pathological characteristics of CHB patients with normal ALT is essential for the early identification of CHB-infected individuals requiring treatment.

Non-alcoholic fatty liver disease (NAFLD) is not a benign condition and carries a risk of progression to liver cirrhosis or HCC as the disease advances (5). With the continuous improvement in living standards and changes in dietary habits and lifestyle, the global prevalence of NAFLD has been rising to over than 25%, making it a common chronic liver disease (6). As the number of NAFLD patients increases, the incidence of CHB patients with NAFLD is also rising. Data suggest that among CHB patients, the biopsy-proven NAFLD can range from 14 to 30% (7). The steatosis and inflammation caused by NAFLD can accelerate liver disease progression in patients with CHB (8, 9). In this study, we analyzed the clinical and pathological characteristics of CHB patients with normal ALT levels, with or without NAFLD. Our aim is to provide more clinical evidence to accurately identify patients with significant liver injury and to potentially expand the indications for antiviral treatment.

## 2 Materials and methods

### 2.1 Patients

In this retrospective analysis, CHB patients diagnosed at the department of traditional and western medical hepatology in Hebei Medical University Third Hospital from January 2008 to December 2022 were enrolled. The upper limit of normal level (ULN) for ALT was defined as 40 U/L. Inclusion criteria were: (1) hepatitis B surface antigen (HBsAg) or HBV DNA positivity for more than 6 months; (2) no prior antiviral therapy; and (3) normal ALT levels. Exclusion criteria encompassed: (1) co-infection with hepatitis A, C, D, or E viruses; (2) concurrent drug-induced liver injury, autoimmune liver disease, cholestatic hepatitis, or alcoholic hepatitis; (3) diagnosed cirrhosis or progression to HCC; and (4) incomplete data. Based on liver biopsy results, the study subjects were categorized into two groups: CHB alone and CHB with NAFLD.

### 2.2 Data collection

Comprehensive patient data were collected, including gender, age, family history, and prior alcohol consumption history. Laboratory tests encompassed platelet counts (PLT), albumin (ALB), ALT, aspartate aminotransferase (AST), γ-glutamdyl transpeptidase (GGT), total bilirubin (TBil), HBeAg, and HBV DNA levels. Liver biopsies were conducted using standard techniques. The indications for liver biopsies are as follows: 1) staging of known parenchymal liver disease to predict prognosis; 2) instructing on whether to start antiviral therapy based on the histologic staging of inflammation and fibrosis in patients with normal ALT; and 3) diagnosis of other liver diseases other than HBV infection. The liver fibrosis and inflammation were classified into five stages (S0–S4) and grades (G0–G4) in accordance with the Scheuer scoring system (10). Significant liver inflammation was defined as G ≥ 2, while significant liver fibrosis was defined as S ≥ 2. Significant liver injury was determined when G and/or S ≥ 2. The assessment of hepatic steatosis followed the histological feature scoring system designed by the Pathology Committee of the NASH Clinical Research Network (11): grade 0 (< 5%), grade 1 (5–33%), grade 2 (33–66%), grade 3 (> 66%). Two pathologists independently evaluated the results and discussed discrepancies until consensus was reached.

### 2.3 Statistical method

Data analysis was conducted using SPSS 21.0 statistical software. Measurement data are presented as median and interquartile range (P25–P75) for non-normally distributed variables for continuous variables, and frequencies and percentages for categorical variables. Non-normally distributed variables were compared using the Wilcoxon rank-sum test. For comparison between groups of categorical data, we used the Chi-squared test. The receiver operating characteristic (ROC) curve was utilized to assess the diagnostic efficacy of the serum non-invasive model. A *P* < 0.05 was considered statistically significant.

## 3 Result

### 3.1 Baseline characteristics of 391 patients with CHB

As presented in Table 1, in the 391 enrolled patients with normal ALT, 284 patients had CHB alone, while 107 had CHB with NAFLD. It comprised 201 (51.41%) males and 190 (48.59%) females, with a median age of 37.0 years [interquartile range (IQR): 31.0–46.0 years]. A total of 125 patients reported a clear family history of HBV infection. The median HBV DNA level was 4.02 log10 IU/mL (IQR: 2.76–7.15 log10 IU/mL), and the median ALT level was 24 U/L (IQR: 18–32 U/L).

**TABLE 1:** Baseline characteristics of 391 patients with CHB ± NAFLD.

Characteristic		Overall (*n* = 391)	CHB alone (*n* = 284)	CHB + NAFLD (*n* = 107)	*P*
Gender, n (%)	Male	201 (51.41)	138 (48.59)	63 (58.88)	0.070
	Female	190 (48.59)	146 (51.41)	44 (41.12)	
Age (year)		37 (31, 46)	37 (30, 45)	38 (32, 49)	0.058
Family history, n (%)	Yes	125 (31.97)	89 (31.34)	36 (33.64)	0.663
	No	266 (68.03)	195 (68.66)	71 (66.36)	
HBV DNA (log10 IU/m L)		4.00 (2.76, 7.15)	3.99 (2.76, 7.02)	4.32 (2.76, 7.50)	0.923
ALT (U/L)		24 (18, 32)	23 (17, 29)	29 (22, 35)	< 0.001
Histopathological fibrosis stage, n (%)	S ≥ 2	227 (58.06)	155 (54.58)	72 (67.29)	0.023
	S < 2	164 (41.94)	129 (45.42)	35 (32.71)	
Grade of liver histopathological inflammation, n (%)	G ≥ 2	191 (48.85)	120 (42.25)	71 (66.36)	< 0.001
	G < 2	200 (51.15)	164 (57.75)	36 (33.64)	
Inflammation grade combined with fibrosis stage, n (%)	G and (or) S ≥ 2	266 (68.03)	182 (64.08)	84 (78.50)	0.006
	G and S < 2	125 (31.97)	102 (35.92)	23 (21.50)	
Degree of hepatic steatosis, n (%)	1 Points	–	–	65 (60.75)	
	Two points	–	–	27 (25.23)	
	Three points	–	–	15 (14.02)	
Expression of HBcAg, n (%)	Positive	179 (45.78%)	129 (45.42%)	50 (46.73%)	0.817
	Negative	212 (54.22%)	155 (54.58%)	57 (53.27%)	

CHB, chronic hepatitis B; NAFLD, non-alcoholic fatty liver disease; ALT, alanine aminotransferase; G, grade of liver histopathological inflammation; S, stage of liver histopathological fibrosis.

Based on the grade of liver pathological inflammation, 191 (48.85%) patients exhibited G ≥ 2, while 200 (51.15%) presented with G < 2. Regarding histopathological fibrosis stage, 227 (58.06%) patients had S ≥ 2, and 164 (41.94%) had S < 2. The distribution of all CHB patients according to their liver inflammation grades (G) and fibrosis stages (S) was as follows: G and/or S ≥ 2 (266, 68.03%) and G and S < 2 (125, 31.97%).

The ALT levels of CHB patients were significantly lower than those of CHB patients with NAFLD (23 vs. 29 U/L, *P* < 0.001). Furthermore, statistical differences were observed in fibrosis stage and inflammation grade between the two groups (*P* < 0.05). The proportion of patients with G and S ≥ 2 was higher in patients with CHB combined with NAFLD. While no significant differences were observed in sex, age, or HBV-DNA load between the CHB alone group and the CHB with NAFLD group (*P* > 0.05).

### 3.2 Incidence of significant liver injury in CHB patients with different HBeAg status, gender, age, and HBV DNA

**FIGURE 1:**
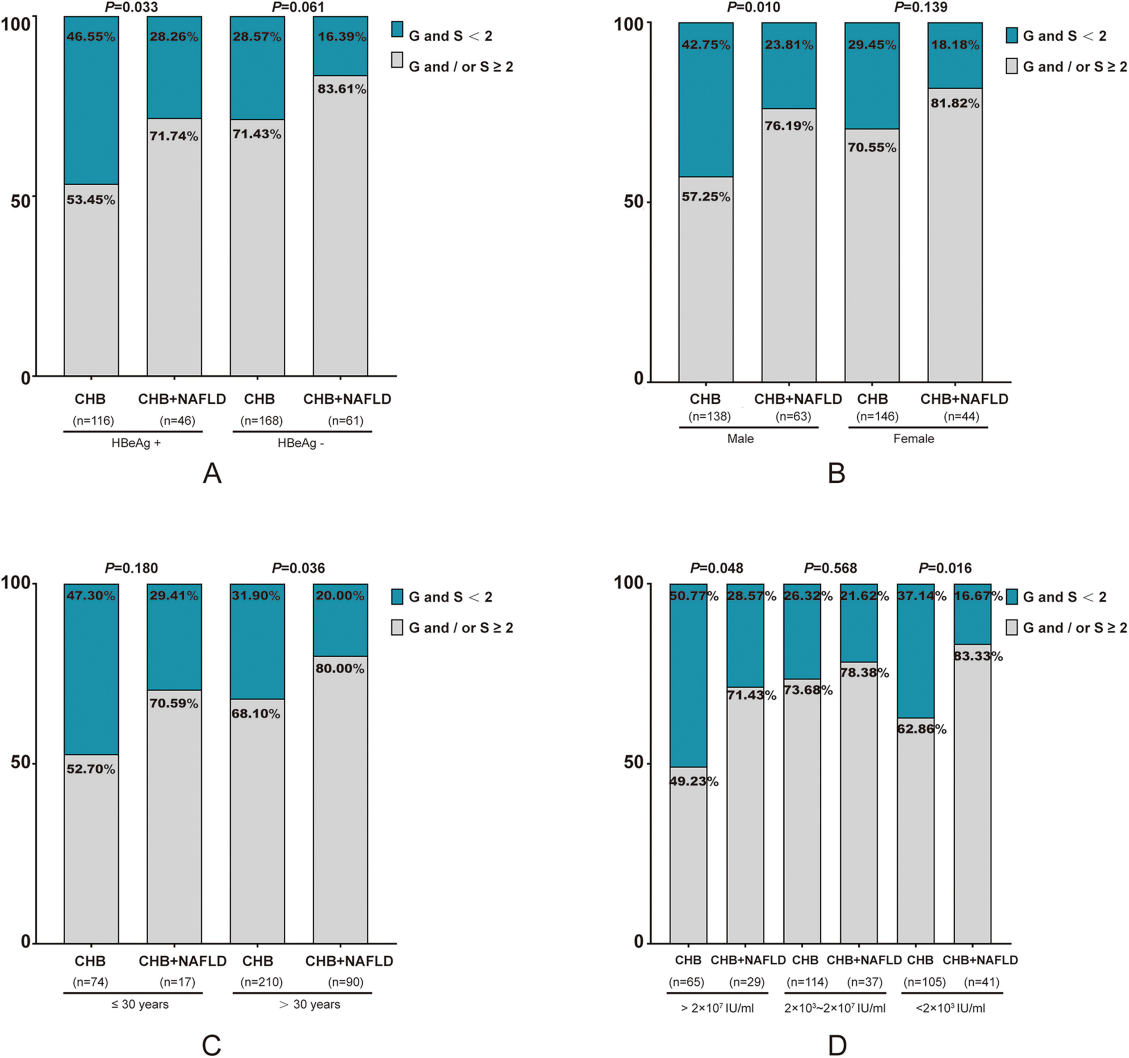
Incidence of significant liver injury in CHB ± NAFLD patients with different HBeAg status, gender, age, and HBV DNA. **(A)** Comparison respectively in patients with positive and negative HBeAg status; **(B)** comparison respectively in males and females; **(C)** comparison respectively in patients with age ≤ 30 years and age > 30 years; **(D)** comparison respectively in patients with HBV DNA > 2 × 10^7^ IU/mL, 2 × 10^3^∼2 × 10^7^ IU/mL and < 2 × 10^3^ IU/mL. CHB, chronic hepatitis B; NAFLD, non-alcoholic fatty liver disease; G, grade of liver histopathological inflammation; S, stage of liver histopathological fibrosis.

As illustrated in Figure 1A, the incidence of significant liver tissue injury in HBeAg positive and negative patients with NAFLD exceeded that of patients with CHB alone (HBeAg positive: 71.74% vs. 53.45%; HBeAg negative: 83.61% vs. 71.43%). However, a statistically significant difference was observed only in the HBeAg positive group (*P* < 0.05).

Irrespective of gender, liver tissue damage in the combined CHB and NAFLD group was more severe than in the CHB alone group, particularly among males, with a statistically significant difference (76.19% vs. 57.25%, *P* < 0.05; Figure 1B).

Irrespective of age beyond 30 years, liver tissue damage in the combined group exhibited greater severity compared to the CHB alone group (age ≤ 30 years: 70.59 vs. 52.70%, *P* > 0.05; age > 30 years: 80.00% vs. 68.10%, *P* < 0.05). This difference was particularly pronounced in patients over 30 years of age (Figure 1C).

Based on viral load levels, the participants were categorized into three groups: high viral load (HBV DNA > 2 × 10^7^ IU/mL), medium viral load (HBV DNA 2 × 10^3^∼2 × 10^7^ IU/mL), and low viral load (HBV DNA < 2 × 10^3^ IU/mL). As illustrated in Figure 1D, both the high and low viral load groups demonstrated a significantly higher incidence of substantial liver tissue damage in CHB patients with NAFLD compared to those with CHB alone (high HBV DNA: 71.43% vs. 49.23%; low HBV DNA: 83.33% vs. 62.86%; all *P* < 0.05). In the moderate viral load group, although CHB patients with NAFLD exhibited a higher incidence of significant liver tissue injury than those with CHB alone (78.38% vs. 73.68%), this difference was not statistically significant (*P* > 0.05).

### 3.3 Relationship between HBcAg expression and HBV-DNA and the degree of inflammation/fibrosis in liver of patients with CHB

Based on the expression of HBcAg in hepatocytes, patients with CHB were categorized into two groups: HBcAg positive and HBcAg negative. Furthermore, the HBcAg positive group was subdivided according to the localization of HBcAg within hepatocytes, resulting in two distinct categories: nuclear expression type and cytoplasmic/cytoplasmic-nuclear expression type.

Among patients diagnosed with CHB alone, HBcAg expression was positive in 129 cases (45.42%) and negative in 155 cases (54.58%), while among patients with CHB combination with NAFLD, HBcAg expression was positive in 50 cases (46.73%) and negative in 57 cases (53.27%). No significant diffrence was observed (*P* > 0.05) (Table 1).

As demonstrated in Table 2, irrespective of CHB alone or combinded with NAFLD, the proportion of cytoplasmic/cytoplasmic nuclear expression type of HBcAg in hepatocytes was higher in patients with G and/or S ≥ 2 compared to those with G and S < 2 in liver tissues (32.42% vs. 16.67% and 36.91% vs. 21.74%). A statistically significant difference was observed in the expression sites of HBcAg in hepatocytes in both CHB alone and combinded with NAFLD patients (*P* < 0.05).

**TABLE 2:** Association between site of HBcAg expression and pathologic inflammation grade combined with fibrosis stage in patients with CHB ± NAFLD.

HBcAg site of the expression site	CHB alone (*n* = 284)		CHB + NAFLD (*n* = 107)	
	G and/or S ≥ 2 (*n* = 182)	G and S < 2 (*n* = 102)	G and/or S ≥ 2 (*n* = 84)	G and S < 2 (*n* = 23)
Cytoplasmic/cytoplasmic nuclear expression type (%)	59 (32.42)	17 (16.67)	31 (36.91)	5 (21.74)
Nuclear expression type (%)*	17 (9.34)	36 (35.29)	6 (7.14)	8 (34.78)
None (%)	106 (58.24)	49 (48.04)	47 (55.95)	10 (43.48)
χ^2^	30.90		12.30	
*P*	< 0.001		0.002	

*vs Cytoplasmic/cytoplasmic nuclear expression type or none group, *P* < 0.001. CHB, chronic hepatitis B; NAFLD, non-alcoholic fatty liver disease; G, grade of liver histopathological inflammation; S, stage of liver histopathological fibrosis.

As shown in Table 3, HBV DNA levels were significantly higher in patients with positive HBcAg expression compared to those with negative expression [7.09 (4.61, 8.08) log10 IU/mL vs. 3.21 (2.70, 4.04) log10 IU/mL, *P* < 0.001] in patients with CHB alone. The similar trend was expressed in patients with CHB combination with NAFLD [7.19 (4.80, 8.13) log10 IU/mL vs. 3.12 (2.63, 4.00) log10 IU/mL, *P* < 0.001].

**TABLE 3:** Association between expression of HBcAg and HBV DNA level in patients with CHB ± NAFLD.

Groups	HBcAg +	HBcAg-	*P*
CHB alone	7.09 (4.61, 8.08)	3.21 (2.70, 4.04)	<0.001
CHB + NAFLD	7.19 (4.80, 8.13)	3.12 (2.63, 4.00)	<0.001

CHB, chronic hepatitis B; NAFLD, non-alcoholic fatty liver disease.

### 3.4 The diagnostic value of non-invasive diagnostic models in the development of significant liver fibrosis

**FIGURE 2:**
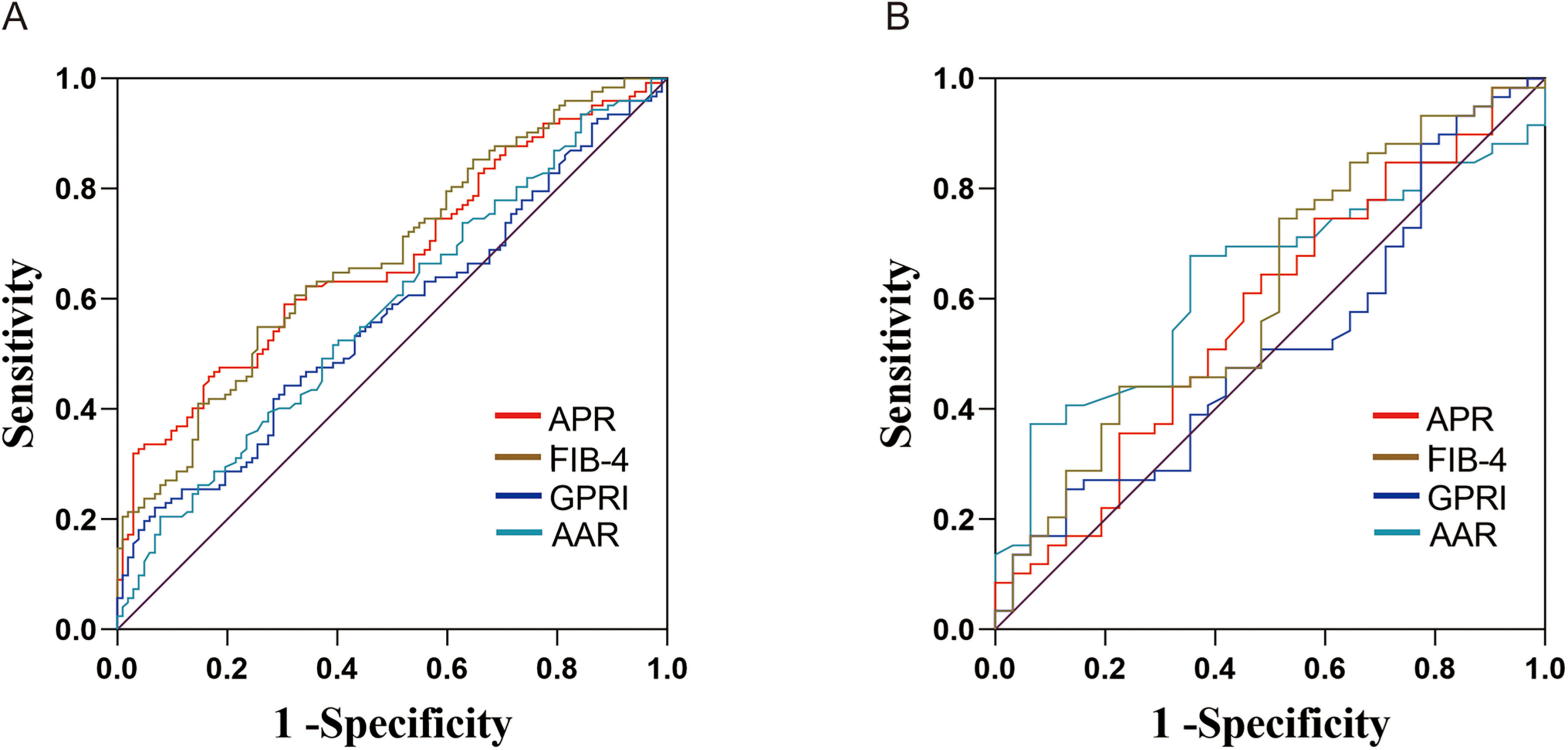
The ROC curves for predicting significant liver fibrosis in patients with CHB alone **(A)** and those with CHB combined with NAFLD **(B)** using non-invasive diagnostic models. CHB, chronic hepatitis B; NAFLD, non-alcoholic fatty liver disease; APRI, aspartate aminotransferase-to-platelet ratio index; FIB-4, fibrosis-4; GPRI, gamma-glutamyl transpeptidase-to-platelet ratio index; AAR, aspartate aminotransferase to alanine aminotransferase ratio index.

For patients with CHB alone, as presented in Table 4 and Figure 2A, the fibrosis-4 (FIB-4) index in the serum non-invasive diagnosis model demonstrated relatively superior efficacy in diagnosing significant liver fibrosis. The area under the receiver operating characteristic curve (AUC) was 0.677, with a 95% confidence interval (CI) of 0.61–0.75. The sensitivity and specificity were 54.9 and 74.5%, respectively.

**TABLE 4:** Diagnostic value of the non-invasive diagnostic model for developing significant liver fibrosis in patients with CHB alone.

Model	AUC (95%CI)	Youden index	Specificity	Sensitivity	Cutoff value	Standard error	*P*
APRI	0.672 (0.60∼0.74)	0.292	88.3%	45.9%	0.34	0.036	<0.01
FIB-4	0.677 (0.61∼0.75)	0.294	74.5%	54.9%	1.000	0.035	<0.01
GPRI	0.565 (0.49∼0.64)	0.153	93.1%	22.1%	0.267	0.038	0.093
AAR	0.579 (0.50∼0.65)	0.126	92.2%	20.5%	1.490	0.038	0.042

CHB, chronic hepatitis B; APRI, aspartate aminotransferase-to-platelet ratio index; FIB-4, fibrosis-4; GPRI, gamma-glutamyl transpeptidase-to-platelet ratio index; AAR, aspartate aminotransferase to alanine aminotransferase ratio index; AUC, area under the receiver operating characteristic curve.

As shown in Table 5 and Figure 2B, in patients with CHB combination with NAFLD, the AST/ALT ratio (AAR) model demonstrated relatively superior diagnostic efficacy, with an AUC of 0.639 (95% CI: 0.52–0.75). The AAR model exhibited a sensitivity of 69.0% and a specificity of 64.5%.

**TABLE 5:** Diagnostic value of the non-invasive diagnostic model for developing significant liver fibrosis in CHB patients with NAFLD.

Model	AUC (95%CI)	Youden index	Specificity	Sensitivity	Cutoff value	Standard error	*P*
APRI	0.574 (0.45∼0.70)	0.161	41.9%	74.1%	0.232	0.064	0.250
FIB-4	0.613 (0.49∼0.74)	0.225	48.4%	74.1%	0.657	0.063	0.080
GPRI	0.518 (0.39∼0.64)	0.130	87.1%	25.9%	0.257	0.064	0.786
AAR	0.639 (0.52∼0.75)	0.335	64.5%	69.0%	0.785	0.059	0.032

CHB, chronic hepatitis B; NAFLD, non-alcoholic fatty liver disease; APRI, aspartate aminotransferase-to-platelet ratio index; FIB-4, fibrosis-4; GPRI, gamma-glutamyl transpeptidase-to-platelet ratio index; AAR, aspartate aminotransferase to alanine aminotransferase ratio index; AUC, area under the receiver operating characteristic curve.

### 3.5 Liver tissue lesions in CHB patients under different upper limits of ALT normality criteria

As the ALT level with the upper limit set at 30 U/L for male and 19 U/L for female, we analyzed the relationship between ALT level and the degree of inflammation/fibrosis in liver of patients with CHB. As shown in Table 6, among patients with ALT levels below sex-specific thresholds (male < 30 U/L, female < 19 U/L), no significant difference was observed between groups (*P* > 0.05). Even if the upper limit threshold of ALT was lowered, there were still many patients with normal ALT levels who had already developed severe liver inflammation and fibrosis.

**TABLE 6:** Liver tissue lesions in CHB patients under different upper limits of ALT normality criteria.

Characteristic	Inflammation grade combined with fibrosis stage		*P*
	G and S < 2	G and/or S ≥ 2	
Total			0.068
High	52 (41.6%)	137 (51.5%)	
Normal*	73 (58.4%)	129 (48.5%)	
CHB alone			0.378
High	41 (40.2%)	83 (45.6%)	
Normal*	61 (59.8%)	99 (54.4%)	
CHB + NAFLD			0.152
High	11 (47.8%)	54 (64.3%)	
Normal*	12 (52.2%)	30 (35.7%)	

*Normal is defined as ALT < 30 U/L in male, female < 19 U/L in female, otherwise, it is High. CHB, chronic hepatitis B; NAFLD, non-alcoholic fatty liver disease; G, grade of liver histopathological inflammation; S, stage of liver histopathological fibrosis.

## 4 Discussion

ALT primarily resides in the cytoplasm of liver cells and is considered the most sensitive indicator for detecting liver dysfunction. Liver injury results in elevated ALT levels, and persistent inflammation can lead to fibrosis progression. Previously, it was believed that CHB patients with normal ALT had low rates of inflammation and fibrosis. However, ALT levels do not directly reflect the degree of liver tissue fibrosis, and numerous studies have demonstrated that CHB patients with normal ALT may still exhibit significant liver tissue inflammatory lesions. Moreover, changes in living conditions and lifestyle have led to a gradual increase in the number of CHB patients with concurrent NAFLD. Despite this trend, studies on the clinical and pathological indicators of CHB accompanied by NAFLD with normal ALT remain limited. This study aims to analyze the clinical and pathological indicators of CHB patients with or without NAFLD, providing evidence for timely anti-HBV treatment initiation.

This study reveals that CHB patients with NAFLD exhibit a higher incidence of significant inflammation or fibrosis and elevated ALT values in liver tissue compared to patients with CHB alone. These findings suggest that fatty degeneration may exacerbate liver injury in CHB patients. Enomot et al. reported significantly elevated ALT levels in CHB patients with NAFLD (12), which aligns with the results of this study. CHB patients with concurrent lipidosis appear more susceptible to liver tissue damage, potentially due to factors such as insulin resistance, genetic predisposition, lipid toxicity, and alterations in intestinal microbiota (13). Yan Huang et al. reported that the presence of NASH was associated with fibrosis progression among CHB patients with normal ALT (14). Several studies have demonstrated that NAFLD may increase the risk of cirrhosis or HCC in CHB patients compared to those with CHB alone (9, 15, 16).

Our study revealed that concomitant NAFLD increased the incidence of significant liver injury in CHB patients, irrespective of sex, HBeAg status, viral load, and age (whether above 30 years or not). This effect was particularly pronounced in HBeAg-positive, male, and older (> 30 years) CHB patients, as well as those with high or low viral loads. Wang et al., analyzing liver biopsy data from 3,212 CHB patients, identified aging and male sex as independent risk factors for NAFLD in CHB patients (17). The potential of estrogen and its receptors to regulate liver fat metabolism, inhibit NAFLD occurrence, and reduce liver tissue inflammation (18) may explain the increased susceptibility of males to significant liver injury. These findings underscore the importance of vigilant monitoring of such patients, including timely liver tissue biopsies for comprehensive condition assessment. However, it is important to note that the current study included a limited number of CHB patients with NAFLD, necessitating further research to corroborate these conclusions.

Among patients with CHB in this study, the incidence of S ≥ 2 was 58.06%, G ≥ 2 was 48.85%, while G and/or S ≥ 2 was 68.03%. Previous studies have demonstrated that in CHB patients with normal ALT levels, the incidence of significant liver injury, as determined by pathological biopsy, ranges from 43.58 to 77.80% (19–21). The findings of this study align with these earlier investigations. Although ALT is currently the most direct and sensitive commonly used indicator of liver tissue injury, it is rapidly deactivated after release into the bloodstream and fluctuates, necessitating dynamic monitoring. Furthermore, ALT levels cannot directly reflect the degree of liver fibrosis and are not proportional to the extent of liver inflammation. Consequently, ALT levels alone cannot accurately assess the severity of liver tissue damage.

This study reveals a significant difference in the expression patterns of HBcAg in hepatocytes between CHB patients with and without significant liver injury. Patients with significant liver injury exhibited a higher proportion of cytoplasmic/cytoplasmic nucleolar HBcAg expression, while those without significant injury showed predominantly nuclear expression. HBcAg, primarily found in HBV-infected hepatocytes or Dane particles, serves as a marker for HBV replication. During viral replication, HBcAg can be categorized as nuclear expression type and cytoplasmic/cytoplasmic-nuclear expression type based on its location within hepatocytes. Subcellular localizaton of HBcAg have been found to be related to the activity of liver. Tae Hyeon Kim et al. had shown that the degree of expression of HBcAg in the hepatocyte nucleus may affect viral load, and the degree of expression of HBcAg in the hepatocyte cytoplasm may affect histologic activities of liver disease (22). The highly immunogenic nature of HBcAg can trigger immune cell activation (23). This research suggests that in CHB patients with normal ALT levels, the transition of HBcAg from the nucleus to the cytoplasm may induce significant liver injury. Consequently, for these patients, alongside monitoring biochemical indicators and assessing pathological fibrosis and inflammation, determining the specific location of HBcAg expression in hepatocytes may provide a more accurate prediction of viral replication and potential immune responses. This insight holds substantial implications for developing tailored follow-up treatment strategies in CHB patients.

In this study, the ROC of APRI, FIB-4, GPRI, and AAR models for predicting significant liver fibrosis in patients with CHB alone were 0.672, 0.677, 0.565, and 0.579, respectively. Serum non-invasive diagnostic models can be derived from simple calculations using blood routine, biochemical and other test indicators, and are widely applied in clinical practice. APRI and FIB-4 were initially utilized to evaluate the degree of hepatitis C-related liver fibrosis. Recent research indicates that APRI and FIB-4 can also assess liver fibrosis in CHB patients (24). A systematic review and meta-analysis demonstrated that APRI and FIB-4 were more effective in diagnosing significant liver fibrosis in CHB patients (25). The GPRI diagnostic model comprises PLT and GGT indicators. A retrospective study by Yu et al. revealed that GPRI’s efficacy in predicting significant liver fibrosis in initially treated CHB patients was comparable to FIB-4 and superior to APRI (26). In this study, the suboptimal predictive efficacy of GPRI may be attributed to the low GGT levels of enrolled patients. AAR is also a risk factor for NAFLD and can evaluate the degree of liver fibrosis in patients with steatosis (27). One study reported that the ROC, sensitivity, and specificity of the AAR model for predicting cirrhosis in NAFLD patients were 0.843%, 76.3%, and 82.9%, respectively (28). The present study demonstrated that the AAR model was superior to other models in predicting significant liver fibrosis in CHB patients with NAFLD, with an area under the ROC curve of 0.639, sensitivity of 69.0%, and specificity of 64.5%. The predictive value of these non-invasive models is not very high. It maybe related to the normal ALT levels or normal liver function. Therefore, it indicates that in the population with normal ALT levels, simple non-invasive diagnostic models are difficult to predict the degree of fibrosis. It further elaborates on the necessity of expanding the indications for treatment.

This study adjusted the ALT threshold from the original 40 U/L to 30 U/L (male) and 19 U/L (female). No significant difference was observed between groups. Even if the upper limit threshold of ALT was lowered, there were still many patients with normal ALT levels who had already developed severe liver inflammation and fibrosis. A separate study involving 1,474 patients with initial treatment of CHB alone compared ALT thresholds of 30 U/L (male)/19 U/L (female) and 40 U/L. The proportion of significant liver tissue injury was 36.40 and 40.40%, respectively, with no statistical difference (21). Although these findings suggest no statistical difference in the proportion of patients with significant liver injury in CHB before and after changing the ALT threshold, lowering the ALT threshold for antiviral therapy may still benefit some patients with significant liver tissue injury.

This study is subject to certain limitations. Firstly, its retrospective nature and relatively small sample size may introduce selectivity bias. Secondly, the single-point measurement of ALT values in this study precludes confirmation of sustained normal ALT levels in the included patients. Consequently, long-term follow-up remains necessary to validate these findings. Thirdly, this is a retrospective cohort study, and data on metabolic comorbidities such as body mass index, fasting blood glucose, lipid indicators, and insulin resistance index, were not systematically collected. The simple steatosis and metabolic dysfunction may have diverse effects on CHB patients. Studies (29, 30) have found that the presence of metabolic dysfunction-associated fatty liver disease in patients with CHB was associated with an increased risk for liver-related clinical events and death, while steatohepatitis did not increase the risk of adverse outcomes. Therefore, further strengthening the monitoring of metabolic-related indicators and clarify their impact on HBV need to be developed in future studies.

## 5 Conclusion

The combination of NAFLD will significantly increase the incidence of significant liver injury in patients with CHB, especially in those who are HBeAg positive, male, over 30 years old, and have either high or low HBV DNA levels. A substantial proportion of patients with CHB alone or in combination with NAFLD, despite exhibiting normal ALT levels, demonstrate significant liver tissue damage. This finding underscores the necessity for comprehensive evaluation utilizing multiple indicators and timely intervention.

## Data Availability

The original contributions presented in this study are included in this article/supplementary material, further inquiries can be directed to the corresponding authors.
